# The challenges of implementation of clinical governance in Iran: a meta-synthesis of qualitative studies

**DOI:** 10.1186/s12961-018-0399-5

**Published:** 2019-01-09

**Authors:** Masoud Behzadifar, Nicola Luigi Bragazzi, Morteza Arab-Zozani, Ahad Bakhtiari, Meysam Behzadifar, Tina Beyranvand, Negar Yousefzadeh, Samad Azari, Haniye Sadat Sajadi, Mandana Saki, Maryam Saran, Hasan Abolghasem Gorji

**Affiliations:** 1 Social Determinants of Health Research Center, Lorestan University of Medical Sciences, Khorramabad, Iran; 2 Department of Health Sciences (DISSAL), School of Public Health, University of Genoa, Genoa, Italy; 3 Iranian Center of Excellence in Health Management, Department of Health Services Management, School of Management and Medical Informatics, Tabriz University of Medical Sciences, Tabriz, Iran; 4 Department of Health Economics and Management, School of Public Health, Tehran University of Medical Sciences, Tehran, Iran; 5 Department of Epidemiology, Faculty of Health & Nutrition, Lorestan University of Medical Sciences, Khorramabad, Iran; 6 Department of Health Services Management, School of Health Management and Information Sciences, Iran University of Medical Sciences, Tehran, Iran; 7 Health Management and Economics Research Center, Iran University of Medical Sciences, Tehran, Iran; 8 National Institute of Health Research, Tehran University of Medical Sciences, Tehran, Iran

**Keywords:** Clinical governance, challenges, Iran, meta-synthesis

## Abstract

**Background:**

Policy- and decision-makers seek to improve the quality of care in the health sector and therefore aim to improve quality through appropriate policies. Higher quality of care will satisfy service providers and the public, reduce costs, increase productivity, and lead to better organisational performance. Clinical governance is a method through which management can be improved and made more accountable, and leads to the provision of better quality of care. In November 2009, the Iranian Ministry of Health and Medical Education implemented new clinical guidelines to standardise and improve clinical services as well as to increase efficiency and reduce costs. The purpose of this study was to assess the challenges of implementing clinical governance through a meta-synthesis of qualitative studies published in Iran.

**Methods:**

Ten databases, including ISI/Web of Sciences, PubMed/MEDLINE, Embase, PsycINFO, the Cochrane Library, CINAHL, Scopus, Barakatns, MagIran and the Scientific Information Database, were searched between January 2009 and May 2018. The quality of the included studies was assessed using the Critical Appraisal Skills Programme tool. This study was reported according to the Enhancing Transparency in Reporting the Synthesis of Qualitative Research guidelines. Thematic synthesis was used to analyse the data.

**Results:**

Ten studies were selected and included based on the inclusion/exclusion criteria. In the first stage, 75 items emerged and were coded, and, following comparison and combination of the codes, 32 codes and 8 themes were finally extracted. These themes included health system structure, management, person-power, cultural factors, information and data, resources, education and evaluation.

**Conclusion:**

The findings of the study showed that there exist a variety of challenges for the implementation of clinical governance in Iran. To successfully implement a health policy, its infrastructure needs to be created. Using the views and support of stakeholders can ensure that a policy is well implemented.

**Trial registration:**

CRD42017079077. Dated October 10, 2017.

**Electronic supplementary material:**

The online version of this article (10.1186/s12961-018-0399-5) contains supplementary material, which is available to authorized users.

## Background

Policy- and decision-makers seek to improve the quality of healthcare provisions through the implementation of appropriate policies [1]. The delivery of high-quality services and the improvement of performance are the main challenges of the healthcare system, with governments worldwide making considerable efforts to achieve this ambitious goal [2]. On the other hand, better quality services is also among the requests and needs of the general public [3, 4]. Higher quality of healthcare services will satisfy both service providers and the public, besides curbing costs, increasing productivity, and leading to a better organisational performance [5]. Concerns about the quality and safety of services, increased people’s expectations about the health system and its performance, high costs, as well as medical errors have made policy- and decision-makers adopt a new approach to overcome these issues [6, 7].

One of the quality approaches that can improve the service level is clinical governance, introduced in 1998 by the Ministry of Health in the United Kingdom. This approach is aimed at making management more accountable, and at providing better quality of care. In clinical governance, all stakeholders are actively involved in the continuous improvement of services and delivery of high-quality care within an appropriate environment [6, 7]. Besides the United Kingdom, clinical governance has been implemented in a number of countries, including Indonesia, Canada, Australia and New Zealand, in an attempt to improve services, obtaining positive results [8–11].

Iran, like many countries, is working to provide high-quality care as one of the most important goals of the health sector. In this regard, it is trying to achieve a comprehensive quality management, implementation of clinical guidelines, and internal audits in order to improve the quality of services provided [12]. However, following the implementation of accreditation in hospitals in Iran, a gap arose regarding the provision of quality services, for which policy- and decision-makers implemented clinical governance [13].

In November 2009, under document number 388044, the Iranian Ministry of Health and Medical Education (MOHME) implemented clinical governance to standardise and improve clinical services, increase efficiency and reduce costs [14]. Since then, a team dedicated to clinical governance, which includes experts and qualified members, has been working in all universities across the country. The team has run numerous quality promotion programmes that included goals such as increasing the satisfaction of hospital, clinic and family physicians, raising the motivation of employees and health system officials to provide optimal services, and implementing quality improvement mechanisms [14]. The core of the programme was based on two key points – collective responsibility and comprehensive reform of hospital structures. The first step of the programme was carried out in the form of 30 projects sponsored by WHO, which established a system of clinical governance in two hospitals in Tehran (Shariati and Roozbeh) [15]. This section was under the supervision of the Deputy of Curative Affairs and had seven categories of activities, namely (1) involving patients, (2) risk management, (3) clinical audits, (4) clinical effectiveness, (5) personal development for practice team, (6) personnel management, and (7) proper use of information [16].

The MOHME has asked the medical departments of universities to provide the necessary infrastructure to implement clinical governance in all hospitals [17]. However, implementing any health policy is generally accompanied by specific problems. Recognising and responding to the challenges of any policy programme aid in its improvement and increased efficiency. Therefore, policy- and decision-makers should have a clear view of their policies [18, 19].

Following the implementation of clinical governance in Iran, various qualitative studies were conducted to address its barriers and challenges from the viewpoints of administrators, policy-makers, managers and healthcare workers. The results of these studies show diverse challenges that have led to some problems in the implementation of this policy.

The purpose of the present study was to investigate the challenges of the implementation of clinical governance in Iran through a meta-synthesis of published qualitative studies.

## Methods

This meta-synthesis was registered in the International Prospective Register of Systematic Reviews (PROSPERO) database of the University of York (CRD42017079077). This study has been reported according to the Enhancing Transparency in Reporting the Synthesis of Qualitative Research (ENTREQ) guidelines [20] (reported in Additional file 1).

Systematic reviews, by providing relevant evidence, play an important role in decision-making and represent potentially useful resources for the health sector [21]. Qualitative studies utilise a set of techniques that deal with description and interpretation of social events and processes, and can provide in-depth and objective insights regarding experiences and points of view [22]. The systematic review and meta-synthesis is a process based on the collection of all qualitative studies published on a given subject and their combination/integration. In this regard, it is possible to create new concepts and frameworks or models for that topic [23, 24].

### Search strategy

Review questions and formulation of the search strategy were conducted according to the Sample, Phenomenon of Interest, Design, Evaluation, Research type (SPIDER) mnemonic, which represents an efficient tool for organising a search strategy of qualitative investigations [25]. Table 1 shows the SPIDER elements adopted in the present study.

**Table 1:** Elements of Sample, Phenomenon of Interest, Design, Evaluation, Research type (SPIDER) mnemonic adopted in this review for strategy search

Elements of SPIDER	Elements of SPIDER as applied to current study
S – Sample	Managers, physicians, nurses, policy- and decision-makers, other stakeholders
PI – Phenomenon of interest	Challenges of the full implementation of clinical governance
D – Design	Qualitative studies
E – Evaluation	Perceptions, views, opinions, experiences
R – Research type	Interviews (semi-structured, in-depth, Delphi technique and focus groups)

Ten databases were searched, including international scholarly ones, such as ISI/Web of Sciences, PubMed/MEDLINE, Embase, PsycINFO, the Cochrane Library, CINAHL and Scopus, as well as Iranian bibliographic thesauri like Barakatns, MagIran and the Scientific Information Database. These databases were searched between January 2009 and May 2018. Reference lists of included studies were assessed to find relevant articles. Google Scholar was also searched for grey literature. The following strategy was used: (“challenges” OR “barriers” OR “problems”) AND (“viewpoints” OR “experience” OR “perception”) AND implementation AND (“clinical governance” OR “quality improvement”) AND Iran AND (“qualitative study” OR “qualitative research” OR “qualitative approach”).

### Study inclusion and exclusion criteria

Studies were included if devised as original qualitative research, investigating and collecting the views, experiences, opinions and perceptions of participants through interviews, published in peer-reviewed journals, and written in either Persian or English.

Studies were excluded if not designed as qualitative investigations, but devised as conference abstracts, case reports, case series, letters to editor, editorial commentaries, expert opinion, interventional studies and reviews.

### Study quality assessment

Two authors (MaB, MAZ) independently assessed the quality and validity of the included studies using the Critical Appraisal Skills Programme (CASP) checklist, composed of 10 questions to help make sense of qualitative research [26]. In order to ensure the reliability of the study quality assessment, a third author (NLB) independently replicated and confirmed/validated the results. The CASP tool consists of ten questions that address issues such as goals, participant selection process, data collection, analysis, and the role of researchers in the results and ethical issues associated with the published study. Three replies to questions are possible, namely (1) yes, (2) no, and (3) cannot tell.

### Data extraction

Two authors (NY, TB) independently extracted the following information: surname of the first author, year of publication, geographic location, number of participants, study design and main findings. Any disagreement was resolved through discussion.

### Data synthesis and presentation

Two authors (MaB, HSS) independently synthesised the data. To analyse and pool the findings of the studies, the Thomas and Harden approach based on thematic analysis was used [27]. This approach is one of the most common methods for analysing qualitative studies in meta-syntheses, and consists of three stages. In the first stage, all codes of included studies were assessed and coded according to their meaning and content. The codes were encoded without creating a hierarchical structure, but using a line-to-line procedure. This was not a simple translation, because we were able to add new items by encoding [28]. Secondly, the authors found similarities and differences among the codes, and new codes based on similarities were added to create the final themes. In this phase, a structure of similar codes was created to facilitate the process of extraction of themes. Up to this point, findings were close to the findings of the studies in that the various codes that emerged were still uncombined. In the third stage, based on the insights and judgments of the authors [29], by going through the content of the studies, the thematic analysis was carried out and the final themes were extracted. In case of disagreement between the two authors, issues were resolve by a third author (NLB) who acted as a judge to reach an agreement on the topic. MAXQDA Version 11 software was used.

### Findings

**Fig. 1:**
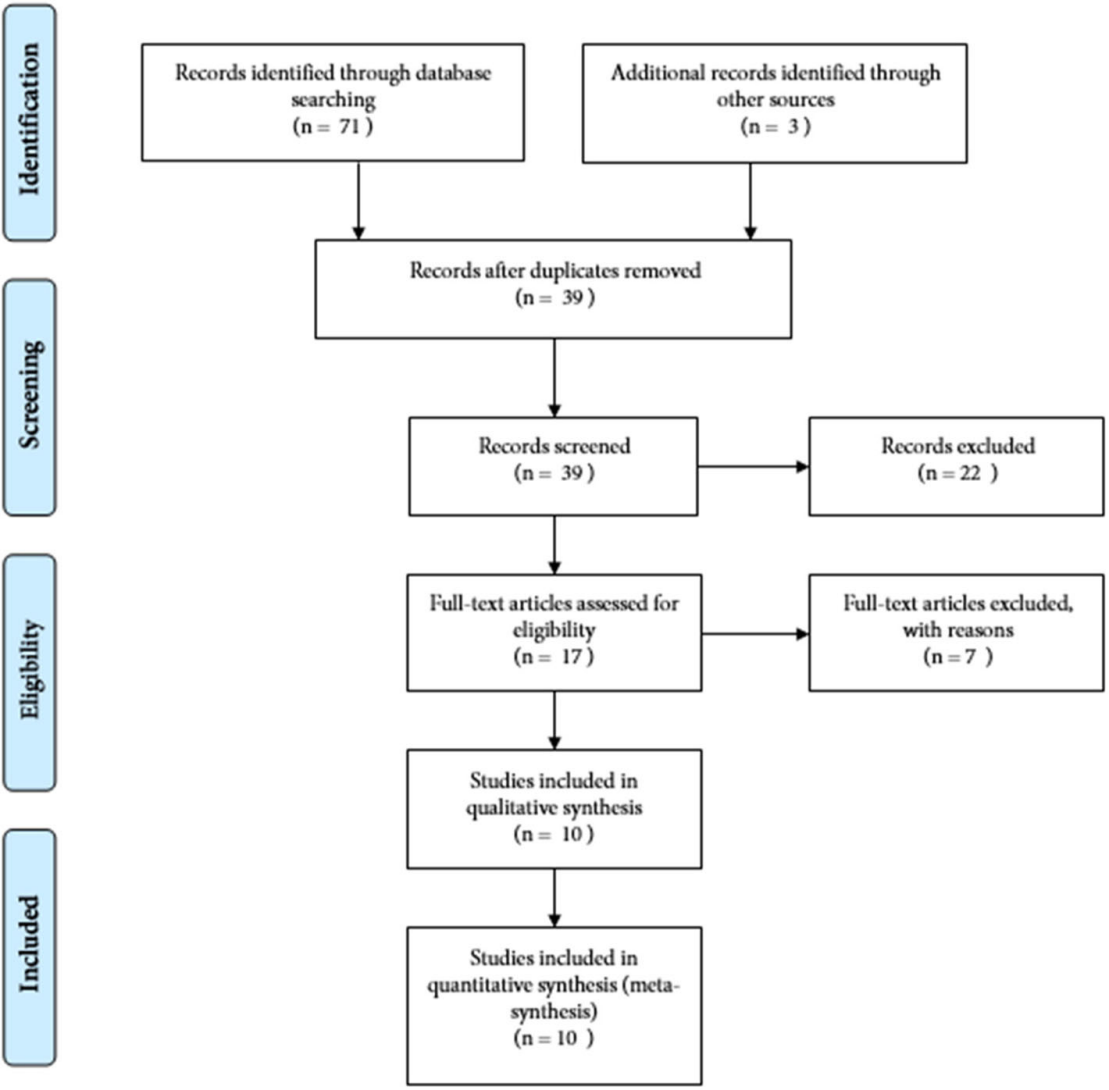
The process of selection of studies

The initial search yielded 74 studies. After the removal of redundant studies, 39 unique investigations were evaluated. A pool of 23 studies were deemed to be non-related to the scope of the meta-synthesis and were therefore removed. The text of 17 studies was read in full and, finally, 10 studies were selected based on the inclusion and exclusion criteria [30–39]. Figure 1 shows the process of searching and selecting studies.

The characteristics of the studies included are shown in Table 2. A total sample of 258 subjects was recruited (range of the study sample size 12–65 individuals), including clinical governance executives and senior managers of teaching hospitals, administrators and clinical staff members (such as nurses, physicians, medical specialists, and laboratory supervisors), and deputies for curative affairs of the Iranian medical universities. Studies were published between 2013 and 2017. Three studies [33, 36, 38] were carried out in the northern part of Iran (Tehran province), while 5 and 2 studies were performed in the centre [30–32, 37, 39] (Isfahan, Kerman and Yazd provinces) and in the west [34, 35] (Qazvin and Tabriz provinces) of Iran, respectively. Eight studies conducted semi-structured interviews, 1 study performed focus groups and the remaining studies performed both semi-structured interviews and focus groups. The length of the interview/focus group ranged from 30 to 120 min. From a methodological standpoint, 4 studies utilised a framework method, 3 studies exploited the thematic analysis and the 3 remaining investigations used the content analysis.

**Table 2:** Characteristics of included studies

First author and reference	Year of publication	Location (city)	Number of participants	Type of participants	Data collection method	Analysis method	Study design
Dehnavieh [30]	2013	Kerman	17	Clinical governance executives of teaching hospitals	Semi-structured interviews, audio recorded, between 55 and 80 min	Framework method	Qualitative
Khayatzadeh-Mahani [31]	2013	Kerman	15	Senior managers at teaching hospitals	Semi-structured interviews and focus group discussion	Thematic analysis	Qualitative
Ataollahi [32]	2014	Yazd	12	Administrators and staff in the hospital treatment sector of Deputy of Treatment and the teaching hospitals	Semi-structured interviews, audio recorded, 45 min	Content analysis	Qualitative
Ravaghi [33]	2014	Tehran	43	Deputies for curative affairs of Iranian medical universities	Semi-structured interviews, audio recorded, 30 min	Thematic analysis	Qualitative
Asefzadeh [34]	2015	Qazvin	17	Senior managers, clinical staff and clinical governance experts	Semi-structured interviews, audio recorded, 44 min	Framework method	Qualitative
Sadeghi-Bazargani [35]	2015	Tabriz	65	Nurses	Focus group discussions, between 90 and 120 min	Content analysis	Qualitative
Ziari [36]	2015	Tehran	25	Nurses, physicians, managers and the personnel of hospitals and Ministry of Health	Semi-structured interviews, audio recorded, between 55 and 84 min	Thematic analysis	Qualitative
Ferdosi [37]	2016	Isfahan	13	Members of health Deputy Clinical Governance office and some hospitals clinical governance team members	Semi-structured interviews, audio recorded, 45 min	Content analysis	Qualitative
Mohaghegh [38]	2016	Tehran	38	Senior managers, medical specialists, nurses, and lab supervisors	Semi-structured interviews, audio recorded, between 45 and 60 min	Framework method	Qualitative
Askari [39]	2017	Yazd	13	Clinical governance executives and deputy members of clinical governance office in curative affairs	Semi-structured interviews, audio recorded	Framework method	Qualitative

**Fig. 2:**
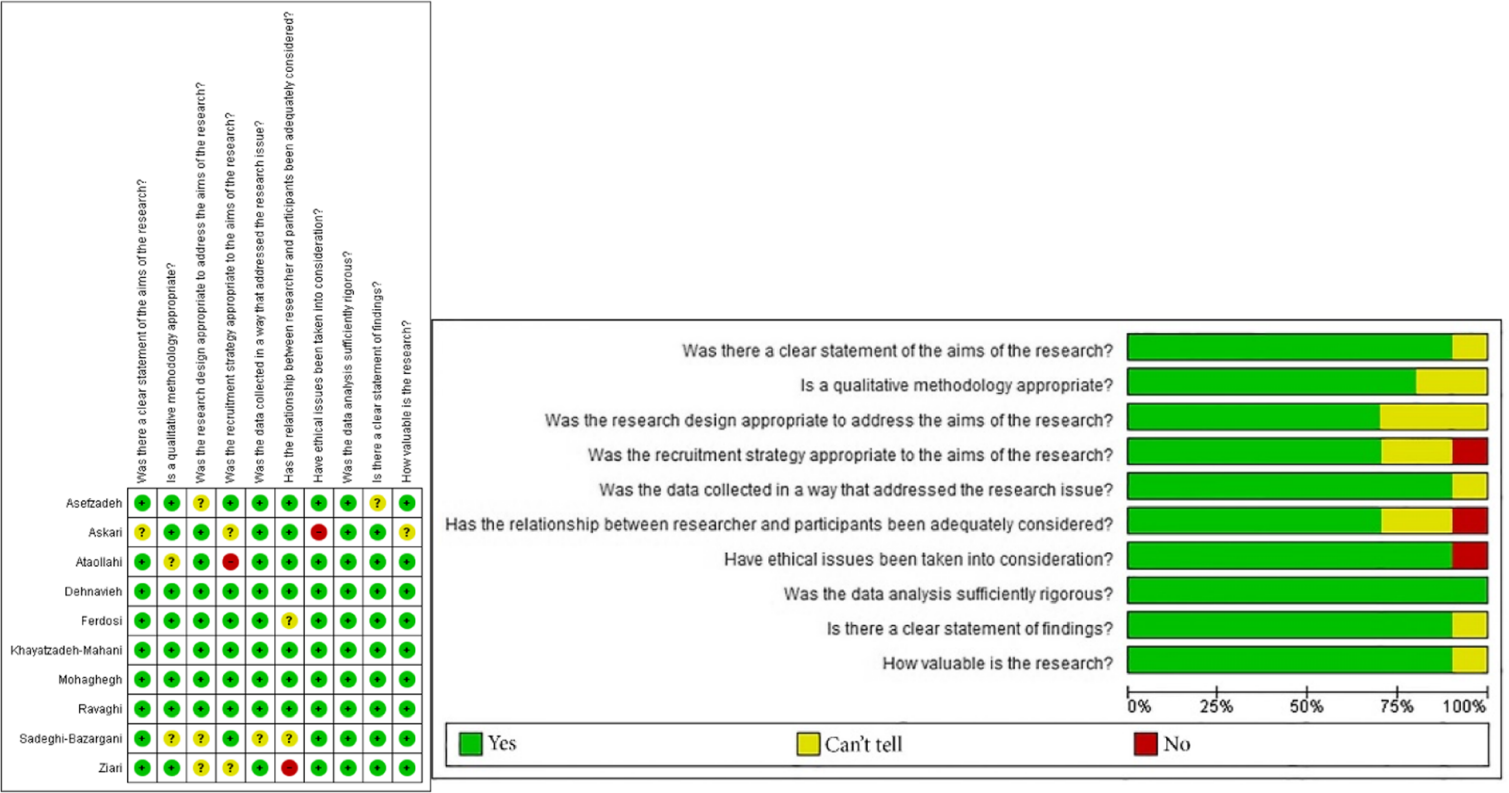
Results of quality assessment

Using the CASP tool, the quality of the studies was evaluated (Fig. 2). More in detail, studies scored from 6 to 10 points (4 studies obtained 10 points, 1 study scored 9, 2 studies reported 8 points, while 1 and 2 studies scored 7 and 6, respectively).

Concerning the thematic analysis, in the first stage, 75 items emerged and were coded, and following comparison of the same codes and their combination, a final set of 32 codes, 8 themes and the most relevant and pertinent quotations were extracted (Table 3).

**Table 3:** The themes and subthemes in this study

Themes	Subthemes	Quotation
Health system structure	Rules related to clinical governance implementation	“*Having an appropriate legislation allows the policy to be implemented in a better and more suitable way. There was a motivation for implementing clinical governance, but there were not many very good laws to guarantee its implementation*” [30]
	Formal structure for clinical governance affairs in the governance arrangement of the health system	“*When clinical governance began, many managers set up a unit to show their interest in running the program, but the unit’s performance was unclear*” [31]
	Inter-sectoral collaboration in the health system	“*The implementation of clinical governance was left to the hospital staff alone. Cooperation with other parts of the Ministry of Health was also needed to implement this program. For example, the support and procurement of some equipment required the cooperation of other deputies*” [33]
	Policy issues	“*Over the time, the Ministry of Health was not interested in the implementation of clinical governance. Financial and manpower* [person-power] *problems have led the managers to pursue other programs*” [31]
Management	Administrator support of clinical governance	“*In the hospitals in which managers were interested in the implementation of clinical governance, they supported activities and employees also had a good incentive to provide services. Good results were obtained*” [36]
	The commitment of managers to clinical governance	“*If they (managers) had the necessary training before running the program, they would surely have had much more support. Employees expected the managers to support the program, but this did not actually happen. Over the time, commitment of managers to run the program has decreased*” [32]
	Planning	“*Having a clear program in mind is very important. The Ministry of Health expects to achieve the goals quickly with the implementation of clinical governance. The* [MOHME Ministry of Health and Medical Education] *should consult all parties to implement the program*” [35]
	Change of managers	“*In Iran, hospital administrators frequently change. The hospital administrators were planning to implement clinical governance as efficiently as possible. It was a matter of time. But as soon as the manager was close to success, it changed, and with the arrival of the new manager, the staff was faced with a new condition for the implementation of clinical governance*” [32]
	Delegation of authority	“*Some managers believe that they are doing things better than others. And it's better to do all the work themselves. They have a lot of tasks and they have not much time to monitor the program. Because of lack of trust in other employees, this has slowed down the activities*” [39]
Person-power	Participation in the implementation of clinical governance	“*Many employees, including physicians, resist against the implementation of the program. They think that the implementation of this program needs many years to achieve its results. They also consider the implementation of clinical governance as opposed to offering their services*” [35]
	Resistance to implementation of clinical governance	“*When a new program should be implemented, a lot of people in all parts of the health sector are opposed to its implementation. Many believe that these programs cannot solve the problems*” [34]
	High workload	“*In addition to my daily activities, I also have services related to clinical governance. I really have no time to do all this and I’m tired*” [33]
Cultural factors	Cultural structures governing the health system of Iran	“*When there is a change to be made, it should be completely clear to everyone. Really, the need for implementation must be clear to the staff. A few months after the program was implemented, many people asked about the tasks and meaning of clinical governance. One of our problems is that there is no consultation with the staff for implementing a program, and the culture of accepting programs is often not provided for staff*” [36]
	Attitude towards clinical governance	“*When serious support is not given to health sector programs, employees do not like it, and they do not make much effort to run programs. Indeed, if the authorities were trying to explain the benefits of this program, then surely the staff would have had a positive attitude toward the program*” [37]
	The role of other stakeholders in the implementation of clinical governance	“*When clinical governance began, many people believed that the implementation of any new program caused more demands from the authorities and, therefore, did not want to cooperate. Moreover, the lack of funding for programs and low motivation has led to a lot of the staff to be strongly critic towards the program*” [36].
	Medical error reporting	“*Many employees are afraid to report medical errors.* *Physicians and nurses, especially physicians, are not likely to report medical errors*” [37].
Information and data	Access to required information	“*With regard to many of the indicators needed to implement clinical governance, we did not have the correct information on the status quo of these indicators. So many programs were not based on reality. The goals that were set were not real*” [30]
	Development of health information system	“*There is no accurate and interconnected hospital information system with adequate equipment; it is not possible to use a variety of fragmentary data to examine the state of implementation of clinical governance programs*” [32]
	Documentation of activities	“*Employees were told that clinical governance activities should be documented. Everyone tried to record the services they were doing. But the equipment was not good for this*” [36]
	Instructions	“*In my opinion, the instructions were very general and ambiguous. If for clinical governance activities the details were correctly stated in the instructions, many of the staff would have been more transparent*” [33]
Resources	Equipment	“*If we want to ensure that clinical governance is implemented in all its dimensions effectively, then there should be various equipment. The hospital was not able to provide all the equipment due to the lack of funds, which greatly affected the correct implementation of the program*” [36]
	Human resources	“*For the implementation of clinical governance, a special human resources unit should be assigned. All hospitals are facing shortage of manpower. Many people, in addition to carrying out activities related to clinical governance, have to do some other work, and therefore their motivation for doing their work is reduced*” [39]
	Financing	“*An adequate budget should be considered for the implementation of this program, and all the activities that the staff members provide should be rewarded. Even for the purchase of some items needed for basic patient safety, there was not enough money*” [30]
Education	Teaching programmes	“*Many hospital managers do not have much knowledge about clinical governance, and because of this, they have little interest in training the rest of the staff. Training classes should be provided before running this program*” [32]
	Clinical governance-related training in medical universities	“*If employees who provide health services in their careers receive training in clinical governance goals, their performance will be better*” [30]
	Patient knowledge and awareness of clinical governance	“*In many hospitals, patients did not know about this program. In some cases, they did not cooperate because they were not aware*” [36]
Evaluation	Evaluation criteria	“*Evaluators need to be scientifically trained to evaluate the performance of the staff in a transparent manner*” [32].
	Issues related to the evaluators	“*Each evaluator has his own criteria and applies his own personal views*” [37]

### Health system structure

#### Rules related to clinical governance implementation

To properly implement a policy, a legislation that facilitates its implementation is needed. Clinical governance was implemented in Iran’s health system with appropriate goals and objectives; however, in many cases, the lack of clear and explicit laws caused executive and administrative problems [31, 33, 37, 38].

#### Formal structure for clinical governance affairs in the governance arrangement of the health system

To better enable the implementation of clinical governance, it is important to define how clinical governance is governed. As such, it would have been of crucial importance to consider the establishment of a formal unit or committee for clinical governance affairs within the MOHME and medical universities and to make all roles and responsibilities clear. Through such structure, employees, with a strong policy and organisational status, would have been more motivated to pay attention to its better implementation due to the use of potential financial benefits and performance enhancement. The absence of such a structure has hindered the full implementation of clinical governance [31, 33, 35, 36, 38].

#### Inter-sectorial collaboration in the health system

Inter-sectorial collaboration is another important issue. To be effective in improving individual and social health, relevant actors, including health professionals, and other parts of the community should collaborate on a range of activities. Many issues indeed require the participation of various governmental and non-governmental sectors. These organisations can all affect health in a variety of ways. An integrated coordination is a key factor in the concept of primary healthcare and health promotion. Our synthesis showed that all measures of implementing clinical governance were only taken by the MOHME and the remaining stakeholders did not actively participate in clinical governance implementation [30, 33, 36, 38].

#### Policy issues

The MOHME, as the main promoter of clinical governance implementation, played a major role in this policy. However, many of the steps necessary for implementing this programme were not considered. Clinical governance was initially put on the agenda of the ministry, but over time, this policy was neglected and overlooked and its implementation failed to improve the quality of its services. In addition, healthcare providers’ protection has been diminished. Sustainability policies were not considered and, in practice, many programmes were not fully implemented. On the other hand, bureaucracy and paperwork played a major role in the process of clinical governance implementation, which caused many people to be discontented. There was also ambiguity in many executive laws, which created confusion among the stakeholders and made the implementation more challenging. Clinical governance was conceived according to a top-down policy perspective, and lower cadre employees were not involved in the achievement of better performance [30, 31, 33, 34, 38].

#### Administrator support to clinical governance

Managers play an important role in implementing programmes. However, hospital managers and other health service providers did not accept the implementation of parallel programmes to improve the quality due to the lack of updated policies and absence of clinical governance as a suitable strategy for promotion [30, 32–34, 36, 38, 39].

#### The commitment of managers to clinical governance

Clinical governance implementation requires an important commitment. The negative attitude of managers impeded them from being actively engaged in the programme. In many cases, they simply communicated the instructions to the employees and did not follow them up [30, 33, 34, 37, 38].

#### Planning

Effective planning for implementing health policy plays a key role in developing and achieving its overall goals. In the planning process, the participation of people involved in the programme in all stages of the design, development, implementation and appraisal of the programme(s) is essential and, for this purpose, identification and communication with the people involved in the programme(s) is important. However, clinical governance planning was carried out by the MOHME alone without collaborations with other stakeholders. Moreover, the whole programme of clinical governance was launched without any pilot implementation, causing considerable inconsistencies during the implementation process [30–33, 36, 37, 39].

#### Change of managers

The rapid change of managers leads to instability and a lack of policy implementation. The new managers, regardless of the performance of the previous administrators and the status of the programme, decided to effectuate major changes in the programme implementation, and thus many employees faced a significant dichotomy in the way the programme was run [30, 32, 36].

#### Delegation of authority

It seemed that delegating the authority of implementing clinical governance aided the better and faster implementation of the programmes. By doing this, a large part of the activities of the executives was performed by staff and other middle managers. However, due to various reasons (e.g. lack of interest in delegation, fear of losing control, lack of trust), the delegation of authority was not fulfilled [32–36, 39].

### Person-power

#### Participation in the implementation of clinical governance

Involving all employees is essential. Their participation creates a framework for their exposure to the programme and a better understanding of the conditions for their services, and leads to improved performance and reinforcement of the workgroup. Unfortunately, regardless of these benefits, some employees (physicians) had no interest or willingness to work in line with clinical governance. Practically, therefore, the group of effective providers to run the programme was limited [30, 32–35, 39].

#### Resistance to implementation of clinical governance

Not believing in clinical governance as a necessity, having different definitions of the need for clinical governance in different parts of the health sector, not believing in the ability to achieve the set goals, not trusting managers and executives, making changes in the programmes, and conflicts of interest of individuals involved are some the examples of resistance to the implementation of the programme [32, 34–36].

#### High workload

The implementation of a new programme, in addition to other programmes and services already being provided by healthcare workers, has increased their workload. On the other hand, the lack of trust and participation of many staff components, especially physicians, led to a high workload for nurses, resulting in their dissatisfaction [30, 32–34, 37].

### Cultural factors

#### Cultural structures governing the health system of Iran

Organisational culture plays an important role in the dynamics of a better implementation of policies in the health sector. The organisational culture in Iran has its own complexity and, therefore, has prevented participation in the implementation of clinical governance. The reform of the health system requires a culture that has the ability to cope with the challenges of the new policy and, if necessary, to demonstrate its flexibility. Therefore, there was no supportive organisational culture to be able to properly implement clinical governance in Iran [30, 32, 36, 37].

#### Attitude towards clinical governance

Unsuccessful implementation of some previous programmes, failure to support policy- and decision-makers, unrealistic expectations about clinical governance goals, ambiguity and inconsistency in the implementation of this policy, and lack of financial funding for employees have prevented staff from having enough incentive to run clinical governance [30, 34–37].

#### The role of other stakeholders in the implementation of clinical governance

Some employees were resistant to the implementation of this programme. This occurred for a variety of reasons, including having different definitions of the need for clinical governance, not believing in the ability to achieve the goals, not trusting managers and executives, making changes to clinical governance, and conflicts of interests of the individuals involved with the programme [31, 33, 36, 37].

#### Medical error reporting

Medical error reporting is important as it is the basis for maintaining and improving patient safety. Despite the ethical and professional commitment of service providers to reveal and disclose cases of error, the reporting rate among health workers is rather low, since they are afraid of the legal penalties [32, 35–37].

### Information and data

#### Access to required information

To properly implement a policy, it is essential to have information about all related issues. Clinical governance implementation in Iran was not properly planned, and there was no information for the staff, people and organisations associated with the programme [30, 32, 36, 37].

#### Development of a health information system

Health information systems lead to improvement and development in accordance with the needs of users and increase the efficiency and effectiveness of hospitals. Having a good health information system helps many employees to increase accuracy, reduces errors and enables the comprehensive monitoring of processes. One of the great health challenges in Iran is the lack of a complete health information system. Although efforts have been made, they have not been sufficient [30, 32, 36, 37].

#### Documentation of activities

Documentation of clinical governance processes is considered an essential step in improving the quality of patient care. Documentation helps to make sure that all efforts to provide better care were properly undertaken by the staff. Unfortunately, due to various reasons, including the lack of proper electronic infrastructure and of electronic records and high workload, most processes were not properly documented [32, 35–37].

#### Instructions

Comprehensive instructions have a valuable role in promoting the processes of clinical governance, and guidelines prepared by the Ministry of Health could significantly help to manage the services provided. However, there was a lack of guidelines for many steps of clinical governance, and employees, in some cases, received ambiguous information about providing clinical governance services and did not know how to behave [32, 33, 36, 37].

### Resources

#### Equipment

The use of physical equipment and infrastructure to implement a policy should be taken into consideration by policy-makers. A proper implementation of clinical governance consists also in the creation of adequate infrastructures for hospitals and other service centres. However, the lack of proper equipment in the health sector has led to problems with the implementation of clinical governance. Additionally, the lack of proper Internet infrastructure, electronic systems, and hospital beds all acted as barriers to the implementation [30, 32–34, 36, 37, 39].

#### Human resources

Good and effective implementation of clinical governance requires adequate human resources. The shortage of nurses and physicians has hindered the implementation of many programmes. The high workload and the attention to the instructions and processes all require sufficient person-power, which should be a priority for policy-makers [30, 32–34, 36, 37, 39].

#### Financing

Dedicated and independent funds for clinical governance in Iran were not considered nor were financial incentives for all employees. Despite the benefits of clinical governance to improve health and reduce the cost of incongruous services, minimal funding was secured for this programme [30, 32, 33, 36, 37, 39].

### Education

#### Teaching programmes

Many service providers were not aware of the importance of clinical governance and there was a lack of knowledge infrastructure for employees. The staff did not complete the necessary training on clinical governance and the retraining classes were also limited. In the face of a lack of financial resources and high costs, managers were not encouraged to hold classes and raise awareness among staff members [30, 32, 37].

#### Clinical governance-related training in medical universities

Most physicians, nurses and other staff members were unfamiliar with clinical governance. In the curricula of Iranian medical universities there were no Clinical Governance courses, which could make future practitioners aware of the goals and impact of such programmes [30, 32].

#### Patient knowledge and awareness of clinical governance

Patients did not know much about clinical governance. Because of their lack of awareness and knowledge, in many cases there was dissatisfaction concerning delivered services. Policy-makers, while planning the implementation of clinical governance, forgot patients as an important part of the programme, and promoted the delivery of services without their cooperation [30, 32, 35–37].

### Evaluation

#### Evaluation criteria

Clinical governance evaluation reduces deviations, and increases the accuracy of operations. The criteria used to evaluate clinical governance in different sectors were in some cases highly unreliable and not standardised, and employees were not able to provide better services due to ambiguities in evaluation criteria [32, 36, 37].

#### Issues related to the evaluators

Failure to properly evaluate clinical governance-related programmes and their implementation status has caused many problems for employees. Lack of evaluators’ knowledge and skills, as well as of consensus among evaluators on how to interpret the results, lack of use of evidence, and of comprehensive guidelines for evaluation led to considerable dissatisfaction among staff members [32, 36, 37].

## Discussion

This is the first and most comprehensive meta-synthesis examining the challenges of clinical governance in the Middle East, and evaluates the quality of clinical governance-related programmes using the results of qualitative studies conducted in Iran. All health systems seek to improve the quality of services provided, and implement different programmes to provide better conditions for their employees and patients.

Based on our thematic analysis, we found eight main themes as challenges for the implementation of clinical governance in Iran.

### Clinical governance and barriers in its implementation

In Iran, the seven-axis clinical governance model, which includes the dimensions of clinical efficacy, clinical audit, risk management, patient and community participation, staffing, education, and information use, was implemented [40]. Based on the findings of the ten included studies, participants believed that the Iranian health sector had attempted to implement clinical governance, yet due to various problems, the goals were not properly achieved. More specifically, clinical governance was not fully implemented due to the lack of guidelines and standardised protocols and the weak organisational culture. Regarding clinical audits, participation of certain groups, such as physicians, was rather low. Because of the high demand for professionals and the real need for services, the work on risk management was impressive. However, despite the high commitment of the MOHME, there were no clear guidelines on this issue. Additionally, there was no extensive training in the field of clinical governance, and the lack of training was particularly felt among the staff. Furthermore, many patients were unfamiliar with this programme. Another issue was the lack of proper development of an information infrastructure and of a suitable platform for using data in clinical governance-related programmes.

### Health system structure

One of the challenges of clinical governance implementation concerned the structure and the status of the health system. Appropriate implementation of policies requires attention and consideration of a series of factors such as equipment, personnel, training, and processes [41–43]. The examination of clinical governance documents indicates the existence of an implementation programme, whereas there was little evidence of the existence of strategies to properly achieve it [31].

The health status of many developing countries such as Iran is still not sustainable due to persistent conflicts between policy- and key decision-makers, decisions that are not evidence informed or based, and the adoption of short-term perspectives, among other factors. As a result, such issues represent serious challenges to the implementation of programmes [18, 44].

### Management

Clinical governance implementation to improve the quality of services provided should be the main goal for staff members. Nevertheless, some employees found the policy goals confusing, ambiguous and inconsistent due to low participation in policy-making process, lack of awareness of the importance of clinical governance and reluctance in participating in programmes that have no clear prospects [45, 46]. The role of physicians can be crucial in achieving the goals of clinical governance [47]; therefore, successfully improving their participation rate seems necessary [48, 49]. Various studies have shown that, due to their negative attitudes and their lack of support to quality programmes, little success has been achieved [50, 51].

The attitudes, values and behaviours of the employees are heavily influenced by the organisational culture, and the management team plays an important role in shaping attitudes towards clinical governance. Hence, the selection and appointment of managers who believe in change and consider clinical governance as an appropriate policy for improving quality will enhance organisational excellence and create a positive attitude towards this policy [52, 53].

An inadequate, non-supportive organisational culture is a major obstacle to achieving a continuous improvement in the quality of healthcare; such an organisational culture may arise due to doubts about the predicted benefits of clinical governance and its consideration as an imposed plan. Such factors could lead to timewasting by staff, inappropriate office formalities, bureaucracy and paperwork, and unnecessary meetings [54].

### Person-power

Despite the fact that a large number of future practitioners are being trained annually, the Iranian Ministry of Health faces a shortage of human resources due to the lack of funds to hire the required people [55]. The lack of sufficient person-power and higher workloads have caused the discontent of many employees, leading to a reduced quality of services provided to patients [56]. The challenge of a shortage of person-power for implementing clinical governance was observed in many Iranian hospitals [33]. Various studies showed that managers need sufficient human resources to establish a proper system of clinical governance [45, 57].

### Cultural factors

Cultural factors were another fundamental challenge in improving the quality of health services [58]. One of the factors of resistance to change is the lack of familiarity with clinical governance programmes [33]. Culture is an important issue in all health sectors, and if we can persuade people, it will play an important role in achieving the goals of improving health services and implementing clinical management [30, 59]. Therefore, in order to implement a policy, cultural infrastructure must first be considered and, with appropriate activities in this regard, we must change the attitudes and behaviour of the people involved in favour of its proper implementation [60].

### Information and data

A good health information system improves performance and decision-making and also plays an important role in delivering patient information and records to service providers in decision-making [61]. It also accelerates the care and treatment processes, improves quality, increases patient satisfaction and reduces costs [62]. One of the key infrastructures for implementing Clinical Governance is the existence of a strong information system for recording and monitoring processes. The problems of inadequate software and hardware, lack of familiarity with the systems and of proper feedback by the system to implement processes were among the issues that made employees unable to take advantage of the new programme [63]. The Ministry of Health has made extensive efforts to create this infrastructure, but this remains insufficient [64].

### Resources

Implementing a policy requires proper facilities, such as person-power, effective equipment and adequate funding [65, 66]. If sustainable resources are not allocated, hospitals will have serious problems to continue service [67]. In this case, wage payment will face long-term delays, and the dissatisfaction among staff members will increase, leading to poor quality of services delivered [68]. The co-operation and support of many people who ensure the implementation of a policy is directly related to appropriate financial resources and therefore adequate and sustainable funding for implementation of a policy needs to be taken into account [69]. The health sector in Iran is heavily dependent on governmental funds. On the other hand, it is not possible for the government to meet all the financial needs of this sector, and therefore the lack of financial resources for implementing clinical governance has created many issues [66].

### Education

Training is appropriate for all people involved in the implementation of clinical management [65]. However, health workers received little training on this policy. Employees were complaining of a lack of proper knowledge [30, 33]. Patients also had little information on clinical governance, and therefore awareness among individuals was not appropriate [32, 38]. Education related to the quality and safety of health services can have a good impact on its improvement [70]. For clinical governance, students in medical universities need to receive the necessary training in this regard.

### Evaluation

The lack of appropriate frameworks for evaluating policies has created many issues for employees involved in clinical governance programmes. In a study by Mohaghegh et al. [38], problem solving, proper examination of documentation, observation of clinical practice and constructive feedback by evaluators were demonstrated to have a good effect in improving the quality of services. On the other hand, the composition of the appraisal team requires major changes, and based on the views of many employees, the evaluation should be performed by a team with a good understanding of clinical governance issues and good management skills. In some studies, the use of teams including physicians, nurses and other healthcare professionals has been emphasised as necessary for a proper evaluation [61, 71].

### Comparison with other studies

Our findings are comparable with those of other available studies [56, 72–74] addressing the challenges of clinical governance implementation in other countries, such as the United Kingdom. According to these studies, barriers to a proper implementation of clinical governance-related programmes are (1) inadequate organisational culture, resistant to change and with poor support from management, (2) negative attitudes of employees, (3) inadequate understanding, insufficient skills and knowledge, (4) lack of time and high workload, (5) lack of adequate funding and resources, and (6) lack of access to information due to inadequate information technology systems.

### Strengths and limitations

The present investigation has some strengths, including the a priori registered study protocol, its methodological rigor, transparency and reproducibility, and the systematic and comprehensive literature search carried out on ten scholarly databases.

However, this study suffers from some limitations, including the fact that assessment of the challenges of the clinical governance implementation was not performed in many Iranian provinces. Furthermore, few studies were conducted on patients’ views.

## Conclusion

This meta-synthesis was conducted to dissect the challenges of implementing clinical governance in Iran in order to provide policy- and decision-makers with an updated, objective synthesis. Recognising and responding to these challenges can help them to better implement healthcare policies. Clinical governance can play an important role in improving the quality of services delivered to patients, and service providers can also better assess their services. Working in a safe and high-quality system for providers is also a good incentive. Raising awareness of managers and staff, making them more supportive to policies, and providing education to all stakeholders involved as well as patients can make this policy plan more effective in Iran.

## Additional file

Additional file 1: Enhancing Transparency in Reporting the Synthesis of Qualitative Research (ENTREQ checklist) (DOCX 21 kb)

## Abbreviations

CASP: Critical Appraisal Skills Programme
MOHME: Ministry of Health and Medical Education
SPIDER: Sample, Phenomenon of Interest, Design, Evaluation, Research type

## References

[1] Dodwad SS (2013). Quality management in healthcare. Indian J Public Health.

[2] Roncarolo F, Boivin A, Denis J-L, Hébert R, Lehoux P (2017). What do we know about the needs and challenges of health systems? A scoping review of the international literature. BMC Health Serv Res.

[3] Veenstra GL, Ahaus K, Welker GA, Heineman E, van der Laan MJ, Muntinghe FLH (2017). Rethinking clinical governance: healthcare professionals’ views: a delphi study. BMJ Open.

[4] Mosadeghrad AM (2012). A conceptual framework for quality of care. Mater Sociomed.

[5] Dilley JA, Bekemeier B, Harris JR (2012). Quality improvement interventions in public health systems: a systematic review. Am J Prev Med.

[6] Gauld R, Horsburgh S (2015). Healthcare professionals’ perceptions of clinical governance implementation: a qualitative New Zealand study of 3205 open-ended survey comments. BMJ Open.

[7] Sarchielli G, De Plato G, Cavalli M, Albertini S, Nonni I, Bencivenni L (2016). Is medical perspective on clinical governance practices associated with clinical units’ performance and mortality? A cross-sectional study through a record-linkage procedure. SAGE Open Med.

[8] Amelia D, Suhowatsky S, Baharuddin M, Tholandi M, Hyre A, Sethi R (2015). Case Study: clinical governance as an approach to improve maternal and newborn health in 22 hospitals in Indonesia. World Health Popul.

[9] Brault I, Denis JL, Sullivan TJ (2015). Using clinical governance levers to support change in a cancer care reform. J Health Organ Manag.

[10] Halton K, Hall L, Gardner A, MacBeth D, Mitchell BG (2017). Exploring the context for effective clinical governance in infection control. Am J Infect Control.

[11] Robertson-Steel I, Edwards S, Gough M (2001). Clinical governance in pre-hospital care. J R Soc Med.

[12] Heyrani A, Maleki M, Marnani AB, Ravaghi H, Sedaghat M, Jabbari M (2012). Clinical governance implementation in a selected teaching emergency department: a systems approach. Implement Sci.

[13] Nekoei-Moghadam M, Amiresmaili M, Iranemansh M, Iranmanesh M (2018). Hospital accreditation in Iran: a qualitative case study of kerman hospitals. Int J Health Plann Manag.

[14] Ravaghi H, Mohseni M, Rafiei S, Shaarbafchi Zadeh N, Mostofian F, Heidarpoor P (2014). Clinical governance in Iran: theory to practice. Procedia Soc Behav Sci.

[15] Ebadi Fardazar F, Safari H, Habibi F, Akbari Haghighi F, Rezapour (2015). Hospitals’ readiness to implement clinical governance. Int J Health Policy Manag.

[16] Mosadeghrad AM, Arab M, Shahidi SN (2017). A survey of clinical governance success in Tehran hospitals. Hakim Health Sys Res.

[17] Ravaghi H, Heidarpour P, Mohseni M, Rafiei S (2013). Senior managers’ viewpoint toward challenges in implementing CG: A national study in Iran. Int J Health Policy Manag..

[18] Walt G, Shiffman J, Schneider H, Murray SF, Brugha R, Gilson L (2008). ‘Doing’ health policy analysis: methodological and conceptual reflections and challenges. Health Policy Plan.

[19] Weeramanthri TS, Bailie RS (2015). Grand challenges in public health policy. Front Public Health.

[20] Tong A, Flemming K, McInnes E, Oliver S, Craig J (2012). Enhancing transparency in reporting the synthesis of qualitative research: ENTREQ. BMC Med Res Methodol.

[21] Lavis JN, Davies HT, Gruen RL, Walshe K, Farquhar CM (2006). Working within and beyond the Cochrane Collaboration to make systematic reviews more useful to healthcare managers and policy makers. Healthc Policy.

[22] Chafe R (2017). The value of qualitative description in health services and policy research. Healthc Policy.

[23] Walsh D, Downe S (2005). Meta-synthesis method for qualitative research: a literature review. J Adv Nurs.

[24] Mohammed MA, Moles RJ, Chen TF (2016). Meta-synthesis of qualitativeresearch: the challenges and opportunities. Int J Clin Pharm.

[25] Cooke A, Smith D, Booth A (2012). Beyond PICO: the SPIDER tool for qualitative evidence synthesis. Qual Health Res.

[26] Critical Appraisal Skills Programme (CASP) (2017). CASP Qualitative Research Checklist.

[27] Thomas J, Harden A (2008). Methods for the thematic synthesis of qualitative research in systematic reviews. BMC Med Res Methodol.

[28] Doyle LH (2003). Synthesis through meta-ethnography: paradoxes, enhancements, and possibilities. Qual Res.

[29] Campbell R, Pound P, Pope C, Britten N, Pill R, Morgan M (2003). Evaluating meta-ethnography: a synthesis of qualitative research on lay experiences of diabetes and diabetes care. Soc Sci Med.

[30] Dehnavieh R, Ebrahimipour H, Jafari Zadeh M, Dianat M, Noori Hekmat S, Mehrolhassani MH (2013). Clinical governance: the challenges of implementation in Iran. Int J Hosp Res.

[31] Khayatzadeh-Mahani A, Nekoei-MoghadamM EA, Ramezani F, Parva S (2013). Clinical governance implementation: a developing country perspective. Clin Governance Int J.

[32] Ataollahi F, Bahrami MA, Atashbahar O, Rejalian F, Gharaie H, Homayooni A (2014). Clinical governance implementation challenges in teaching hospitals affiliated to Yazd University of Medical Sciences, Iran, based on the experts’ viewpoint. J Manage Med Inform Sch.

[33] Ravaghi H, Rafiei S, Heidarpour P, Mohseni M (2014). Facilitators and barriers to implementing clinical governance: a qualitative study among senior managers in Iran. Iran J Public Health.

[34] Asefzadeh S, Taghizadeh S, Heyrani A, Mohebbifar R, Arabloo J (2015). An exploration of clinical governance implementation and assessment challenges in qazvin teaching hospitals: a qualitative study. Payavard.

[35] Sadeghi-Bazargani H, Tabrizi JS, Saadati M, Hassanzadeh R, Alizadeh G (2015). Nursing experiences of clinical governance implementation: a qualitative study. Clin Governance Int J.

[36] Ziari A, Abachizade K, Rassouli M, Haidarnia MA, Mohseny M (2015). Assessment of barriers of implementation of clinical governance in educational hospitals of Shahid Beheshti University of Medical sciences: a qualitative study. J Hosp.

[37] Ferdosi M, Ziyari FB, Ollahi MN, Salmani AR, Niknam N (2016). Implementing clinical governance in Isfahan hospitals: Barriers and solutions, 2014. J Edu Health Promot.

[38] Mohaghegh B, Ravaghi H, Mannion R, Heidarpoor P, Sajadi HS (2016). Implementing clinical governance in Iranian hospitals: purpose, process and pitfalls. Electron Physician.

[39] Askari R, Dolatian M, Shafil M, Baghian N, Rafiel S (2017). Challenges in implementing clinical governance: A qualitative study in Yazd. Iran East Afr Med J.

[40] McCormick S, Wardrope J, Perez AC (2002). Quality assurance clinical governance, and a patient wants to die. Emerg Med J.

[41] Hartley AM, Griffiths RK, Saunders KL (2002). An evaluation of clinical governance in the public health departments of the West Midland Region. J Epidemiol Community Health.

[42] Hagan H, Basnett I, McKee M (2007). Consultant’s attitudes to clinical governance: barriers and incentives to engagement. Public Health.

[43] Balding C (2005). Strengthening clinical governance through cultivating the line management role. Aust Health Rev.

[44] Watt S, Sword W, Krueger P (2005). Implementation of a health care policy: an analysis of barriers and facilitators to practice change. BMC Health Serv Res.

[45] Som CV (2007). Exploring the human resource implications of clinical governance. Health Policy.

[46] Som CV (2009). Sense making of clinical governance at different levels in NHS hospital trusts. Clin Governance Int J.

[47] Ham C, Kipping R, McLeod H (2003). Redesigning work processes in health care: lessons from the national health service. Milbank Q.

[48] Weiner BJ, Shortell SM, Alexander J (1997). Promoting clinical involvement in hospital quality improvement efforts: the effects of top management, board, and physician leadership. Health Serv Res.

[49] Blumenthal D, Kilo CM (1998). A report card on continuous quality improvement. Milbank Q.

[50] Spooner A, Chapple A, Roland M (2001). What makes British general practitioners take part in a quality improvement scheme?. J Health Serv Res Policy.

[51] Walshe K, Freeman T (2002). Effectiveness of quality improvement: learning from evaluations. Qual Saf Health Care.

[52] Prenestini A, Calciolari S, Lega F, Grilli R (2015). The relationship between senior management team culture and clinical governance: empirical investigation and managerial implications. Health Care Manag Rev.

[53] Marshall M, Sheaff R, Rogers A, Campbell S, Halliwell S, Pickard S (2002). A qualitative study of the cultural changes in primary care organisations needed to implement clinical governance. Br J Gen Pract.

[54] Walshe K, Chambers N. Clinical governance and the role of NHS boards: learning lessons from the case of Ian Paterson. BMJ. 2017;357:j2138. 10.1136/bmj.j2138 28465310

[55] Lotfi F, Bayati M, Yusefi AR, Ghaderi S, Barati O (2018). Inequality in distribution of health care resources in Iran: human resources, health centers and hospital beds. Shiraz E-Med J.

[56] Sweeney GM, Sweeney KG, Greco MJ, Stead JW (2002). Softly, softly, the way forward? A qualitative study of the first year of implementing clinical governance in primary care. Prim Health Care Res Dev.

[57] Som CV (2011). Clinical governance and attention to human resources. Br J Healthc Manag.

[58] Hackett M, Lilford R, Jordan J (1999). Clinical governance: culture, leadership and power--the key to changing attitudes and behaviours in trusts. Int J Health Care Qual Assur Inc Leadersh Health Serv.

[59] Plough AL (2015). Building a culture of health: a critical role for public health services and systems research. Am J Public Health.

[60] Milligan FJ (2007). Establishing a culture for patient safety - the role of education. Nurse Educ Today.

[61] Hooshmand E, Tourani S, Ravaghi H, Ebrahimipour H (2014). Challenges in evaluating clinical governance systems in Iran: a qualitative study. Iran Red Crescent Med J.

[62] Ohno-Machado L (2012). Cost-effectiveness of informatics and health IT: impact on finances and quality of care. J Am Med Inform Assoc.

[63] Jeddi FR, Farzandipoor M, Arabfard M, Hosseini AHM (2014). Conceptual model of clinical governance information system for statistical indicators by using UML in two sample hospitals. Acta Inform Med..

[64] Jahanbakhsh M, Sharifi M, Ayat M (2014). The status of hospital information systems in Iranian hospitals. Acta Inform Med.

[65] Gauld R, Horsburgh S (2014). Measuring progress with clinical governance development in New Zealand: perceptions of senior doctors in 2010 and 2012. BMC Health Serv Res.

[66] Mousavi SM, Agharahimi Z, Daryabeigi M, Rezaei N (2014). Implementation of clinical governance in hospitals: challenges and the keys for success. Acta Med Iran.

[67] Liaropoulos L, Goranitis I (2015). Health care financing and the sustainability of health systems. Int J Equity Health.

[68] Hsiao WC (2007). Why is a systemic view of health financing necessary?. Health Aff.

[69] Walsh K (2014). Clinical governance: costs and benefits. Int J Health Policy Manag.

[70] Starr SR, Kautz JM, Sorita A, Thompson KM, Reed DA, Porter BL (2016). Quality improvement education for health professionals: a systematic review. Am J Med Qual.

[71] Vali L, Mastaneh Z, Mouseli A, Kardanmoghadam V, Kamali S (2017). Success rate evaluation of clinical governance implementation in teaching hospitals in Kerman (Iran) based on nine steps of Karsh’s mode. Electron Physician.

[72] Shakeshaft AM (2008). A study of the attitudes and perceived barriers to undertaking clinical governance activities of dietitians in a Welsh national health service trust. J Hum Nutr Diet.

[73] Latham L, Freeman T, Walshe K, Spurgeon P, Wallace L (2000). Clinical governance in the West Midlands and South West regions: early progress in NHS trusts. J Clin Manag.

[74] Campbell SM, Sheaff R, Sibbald B, Marshall MN, Pickard S, Gask L (2002). Implementing clinical governance in English primary care groups/trusts: reconciling quality improvement and quality assurance. Qual Saf Health Care.

